# Comparing three types of data-driven models for monthly evapotranspiration prediction under heterogeneous climatic conditions

**DOI:** 10.1038/s41598-022-22272-3

**Published:** 2022-10-17

**Authors:** Pouya Aghelpour, Vahid Varshavian, Mehraneh Khodamorad Pour, Zahra Hamedi

**Affiliations:** 1 Department of Water Engineering, Faculty of Agriculture, Bu-Ali Sina University, Hamedan, Iran; 2 Computer Science Department, University of Birmingham, Birmingham, UK

**Keywords:** Climate sciences, Hydrology

## Abstract

Evapotranspiration is one of the most important hydro-climatological components which directly affects agricultural productions. Therefore, its forecasting is critical for water managers and irrigation planners. In this study, adaptive neuro-fuzzy inference system (ANFIS) model has been hybridized by differential evolution (DE) optimization algorithm as a novel approach to forecast monthly reference evapotranspiration (ET0). Furthermore, this model has been compared with the classic stochastic time series model. For this, the ET0 rates were calculated on a monthly scale during 1995–2018, based on FAO-56 Penman–Monteith equation and meteorological data including minimum air temperature, maximum air temperature, mean air temperature, minimum relative humidity, maximum relative humidity & sunshine duration. The investigation was performed on 6 stations in different climates of Iran, including Bandar Anzali & Ramsar (per-humid), Gharakhil (sub-humid), Shiraz (semi-arid), Ahwaz (arid), and Yazd (extra-arid). The models’ performances were evaluated by the criteria percent bias (PB), root mean squared error (RMSE), normalized RMSE (NRMSE), and Nash-Sutcliff (NS) coefficient. Surveys confirm the high capability of the hybrid ANFIS-DE model in monthly ET0 forecasting; so that the DE algorithm was able to improve the accuracy of ANFIS, by 16% on average. Seasonal autoregressive integrated moving average (SARIMA) was the most suitable pattern among the time series stochastic models and superior to its competitors, ANFIS and ANFIS-DE. Consequently, the SARIMA was suggested more appropriate for monthly ET0 forecasting in all the climates, due to its simplicity and parsimony. Comparison between the different climates confirmed that the climate type significantly affects the forecasting accuracies: it’s revealed that all the models work better in extra-arid, arid and semi-arid climates, than the humid and per-humid areas.

## Introduction

The process of water parting the surface of moist soil is called evaporation, whereas this phenomenon from leaves’ pores is called transpiration. Since recognizing these two phenomena on farms is not easy, they are considered one single integrated variable referred to as “evapotranspiration.” Since evapotranspiration is defined on the surface of an agricultural land, it also includes the water deposited by rain, irrigation, or dew drops on leaves. On the other hand, evapotranspiration is regarded as the water requirement of plants; thus, its measurement is essential in all agricultural and irrigation projects. The amount of evapotranspiration is measured by a lysimeter. Due to the sensitivity of the lysimeter, a technician expert is needed on-site to calibrate the lysimeter constantly. Consequently, if the recorded cases of lysimeter are not cared for carefully, they may have errors. As a remedy, the International Commission on Irrigation and Drainage (ICID) and World Meteorological Organization (WMO) have recognized that the FAO-56 Penman–Monteith equation (FAO-56 PM) should be an acceptable substitute for scarce lysimeter data (Allen et al.^1^).

In recent years, despite some well-known mathematical models such as Penman–Monteith, Thornthwaite, Hargreaves-Samani, Blaney-Criddle, etc., the black-box artificial intelligence (AI) models have shown acceptable accuracy in estimating evapotranspiration. For example, Mohammadi and Mehdizadeh^2^ and Ahmadi et al.^3^, surveying the arid and semi-arid regions of Iran, found that the AI models can estimate evapotranspiration with reasonable accuracy and the least available meteorological variables in the complete absence of meteorological variables, which are required to use the Penman method. They also contended that integrating AI models with bio-inspired optimization algorithms can significantly increase the accuracy of evapotranspiration estimation. In Australia, AIs could accurately estimate evapotranspiration with only temperature and wind speed as available variables (Falamarzi et al.^4^) that can be considered a suitable alternative for the FAO-56 PM model when the meteorological variables are missing. Also, in cases such as Kumar et al.^5^, lysimeter measured evapotranspiration values were used for the validation of the estimated evapotranspiration from neural networks, and their comparison with the outputs of the FAO-56 PM model showed that AIs could be a better estimator for evapotranspiration.

Reference evapotranspiration (ET0) is one of the main components of the hydrological cycle associated with agricultural systems. Accurate estimation and prediction of ET0 are critical in water resources management, irrigation planning, and determining plants’ water needs. Forecasting the ET0 rates by providing information on the future status of evapotranspiration at different time scales can help make appropriate decisions, plan, and apply management methods of water resources. The information for the next day(s), for short-term decisions and planning will be provided on a daily scale prediction. On a monthly scale prediction of ET0, obtaining a longer-term perspective of ET0 changes in the future is possible, which will be especially useful for crops with a long-term growth period (several months). Also, evaluating the agricultural drought status, which is done by famous indicators such as standardized precipitation-evapotranspiration index (SPEI) and Palmer drought severity index (PDSI), directly requires the monthly scale ET0 rate of the region. Data-driven models like stochastic and artificial intelligence methods are efficient approaches that have shown good performance in modeling and predicting hydrometeorological variables in recent years (Essam et al.^6^; Dehghanisanij et al.^7^; Elbeltagi et al.^8^; Azad et al.^9^; Zhang et al.^10^; Zarei et al.^11^; Graf and Aghelpour^12^; Chen et al.^13^). In ET0 cases, Karbasi^14^ have used AIs for ET0 forecasting in 1, 2, 3, 7, 10, 14, 18, 24, and 30 days lead times. Karbasi^14^ concluded that the accuracy of the predictions was desirable and showed that when the forecast horizon increases, the forecasting accuracy decreases. A comparison between stochastic and artificial intelligence methods in Spain revealed that both model types predicted weekly evapotranspiration effectively (Landeras et al.^15^). Lucas et al.^16^ compared the seasonal autoregressive integrated moving average (SARIMA) stochastic model with the convolutional neural network (CNN) model to predict daily evapotranspiration in Brazil. They concluded that the CNN model can provide a more accurate prediction of evapotranspiration than the SARIMA model. In contrast, in the Tamil Nadu of India, a comparison was made between artificial intelligence and stochastic methods, and then more appropriate stochastic models were introduced for predicting ET0 (Kishore and Pushpalatha^17^).

Predicting evapotranspiration, especially in areas like Iran, which are facing limited water resources, is doubly crucial for determining the cultivation pattern and proper management of water and soil resources. In Iran, these two types of numerical models, i.e., stochastics and AIs, have been used to predict ET0. Ashrafzadeh et al.^18^ used the SARIMA, group method of data handling (GMDH), and support vector machine (SVM) models to predict ET0 in humid areas of the Caspian Sea’s southern margin (Guilan province). They evaluated the accuracy of the models and indicated that the mentioned models can predict the ET0 value for the next two years, with the same suitable accuracy as the train-test period. In the same region in Iran (Mazandaran province), Aghelpour and Norooz-Valashedi^19^ compared these two model types for the daily prediction of ET0 rates. They applied the models’ autoregressive (AR), moving average (MA), autoregressive moving average (ARMA) and autoregressive integrated moving average (ARIMA) as stochastic models, and compared them with three AIs including SVM, generalized regression neural network (GRNN), and adaptive neuro-fuzzy inference system (ANFIS). The results have shown the high capability of both model types in predicting daily ET0 rates for this humid region. Also another study has developed by Aghelpour et al.^20^ for the estimation (not prediction) of rice evapotranspiration in this region. They have found that the AIs like GMDH, GRNN, multilayer perceptron (MLP), and radial basis function neural network (GRNN) are capable of providing a high accuracy estimation for the daily evapotranspiration rates of rice crop, which is the most important agricultural crop of this region.

The combination of bio-inspired optimization algorithms has significantly improved the AI performance of AIs in most cases (Ahmadianfar et al.^21^; Mehdizadeh et al.^22^; Ahmadi et al.^3^; Babanezhad et al.^23^; Mohammadi et al.^24^; Aghelpour and Varshavian^25^; Deo et al.^26^). These algorithms that use complex evolutionary methods can optimally enhance the parameters of AIs and significantly increase the accuracy of the estimations and predictions. In the AIs, the parameter optimization process is commonly done by the linear least square or gradient decent algorithms, which may suffer from the local optimum problem. To dominate this problem, bio-inspired optimizers, which use nature-inspired search procedures rather than derivatives to find optimal solutions, are suggested in some studies to train AIs. Since there are many natural sources of inspiration, a host of bio-inspired optimizers can be found in the literature. However, just a few of these algorithms have been used in ET0 prediction cases. For example, Mohammadi and Mehdizadeh^2^ have shown that in daily evapotranspiration modeling, a bio-inspired algorithm like the whale optimization algorithm can improve the accuracy of AIs in modeling reference evapotranspiration rates. Genetic and firefly are two other well-known bio-inspired algorithms that have significantly increased the AIs’ accuracy in evapotranspiration modeling cases (Roy et al.^27^; Tao et al.^28^; Eslamian et al.^29^; Aghajanloo et al.^30^; Yin et al.^31^; Gocić et al.^32^). Differential evolution (DE) is another bio-inspired optimization algorithm that has been less used in this term. For example, it was well evaluated to improve the AIs’ accuracy in some cases, such as solar radiation estimation (Babatunde et al.^33^; Halabi et al.^34^), pan evaporation modeling (Wu et al.^35^), dust source modeling (Rahmati et al.^36^), or drought prediction (Aghelpour et al.^37^), but has been rarely evaluated in evapotranspiration modeling cases.

The ANFIS model is one of the most efficient AI methods that has been used in both simple and hybridized forms for hydrological and meteorological modeling. ANFIS model showed its acceptable performances in solar radiation estimation (Üstün et al.^38^; Halabi et al.^34^; Khosravi et al.^39^), pan evaporation estimation (Adnan et al.^40^; Guven and Kisi^41^), drought forecasting (Aghelpour et al.^42^; Aghelpour et al.^43^; Aghelpour et al.^44^; Kisi et al.^45^), river flow modeling (Mohammadi et al.^46^; Aghelpour et al.^47^), rainfall forecasting (Mekanik et al.^48^; Yaseen et al.^49^), and wind speed forecasting (Maroufpoor et al.^50^). However, they are less used in evapotranspiration prediction for the future (most of the studied cases have used the ANFIS model for ET0 “estimation,” not “prediction” for the future). The present study intends to use the ANFIS model to predict the reference evapotranspiration and compare it with the classical SARIMA stochastic model. Moreover, as a novelty, the DE algorithm is combined with the ANFIS model as ANFIS-DE in this study to optimize and improve the ANFIS’s prediction accuracy. This research studies stations from different climates (from extra-arid to per-humid). Moreover, investigating the effect of the climate type on the accuracy of the models predicting ET0 for the first time is another novelty aspect of the current research.

## Materials and methods

### Data and areas under investigation

**Figure 1:**
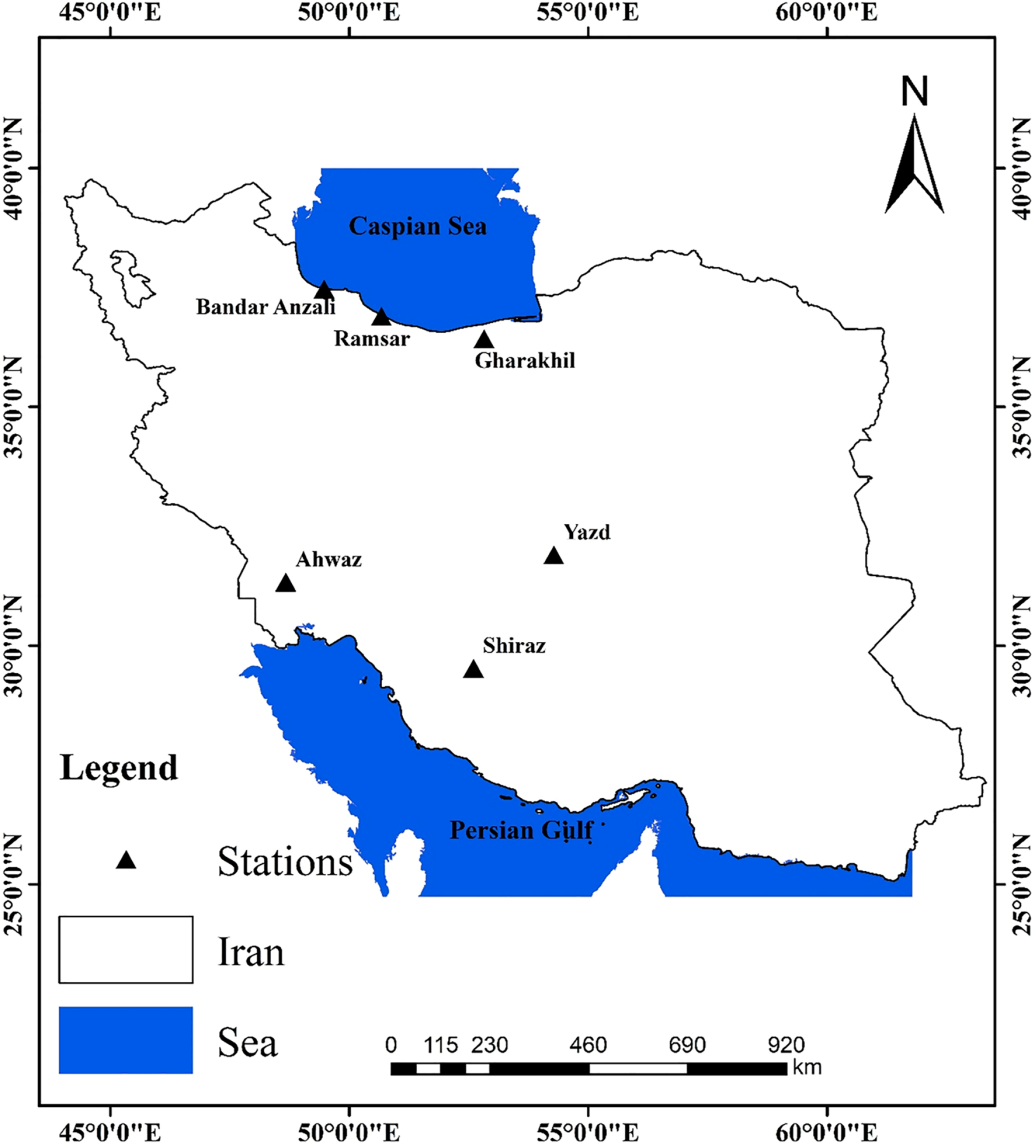
Location of the stations under investigation on the country (the map is generated in R software by the authors).

Iran is located in the Middle East, on the dry belt of the earth. Consequently, it is facing limited water resources in human life’s different sectors, such as agriculture. According to De-Martonne climatic zoning, Iran has 28 different climatic classes (Rahimi et al.^51^). The majority of regions in Iran have arid (central desert, southwest, and southwest of the country) and semi-arid climates (the Zagros Mountains in the west and northwest of the country as well as northeastern regions), and only small areas of Iran have humid climates (the Southern shore of the Caspian Sea in the north). The evapotranspiration rate, which is affected by different meteorological factors, varies in different climatic zones. For example, in arid regions like Ahwaz, the range of ET0 is between 40 and 350 mm per month, while in humid climates like Ramsar, the ET0 varies between 20 and 158 mm per month. This paper aims to investigate the effect of the climate type on the accuracy of models predicting evapotranspiration. For this, six synoptic stations from different climates of Iran are considered, which are illustrated as Fig. 1 (R packages “sf” [Pebesm^52^] and “ggplot2” [Wickham^53^] were used to draw this figure).

Three stations were selected from the humid and sub-humid areas of northern Iran (on the southern margin of the Caspian Sea). The other three stations were from arid and semi-arid areas in central and southwestern parts of Iran. Most of the agricultural lands in the humid northern areas are under rice cultivation, and the horticultural lands in this area are often under citrus cultivation. In the arid and semi-arid regions of the southern parts of Iran, the main crops include wheat and maize, and the important horticultural crops are grapes and pistachios. A summary of the information about this study’s climatic zones, stations, and common products is shown in Table 1.

**Table 1:** The studied stations’ location, climate (according to extended De-Martonne classification) and the main agricultural/horticultural products of their regions.

Province	Station	Coordinates			Climate (based on extended De-Martonne method)	Main products	
		Latitude–northern (degree)	Longitude–eastern (degree)	Elevation (m)		Agricultural	Horticultural
Gilan	Bandar Anzali	37.47	49.47	− 26.2	Per humid(B)—Moderate	Rice cultivars; tobacco; watermelon	Tea; olive; citrus; kiwi; plum
Mazandaran	Ramsar	36.90	50.67	− 20.0	Per humid(A)—Moderate	Rice cultivars; wheat; soy; rapeseed	Citrus; kiwi; ornamental flower; plants
	Gharakhil	36.45	52.77	14.7	Sub-humid—Moderate		
Khuzestan	Ahwaz	31.33	48.67	22.5	Arid—Warm	Wheat; barley; maize; legumes; rapeseed	Vegetable; cucurbits; potato; onion
Fars	Shiraz	29.53	52.60	1484.0	Semi-arid—Moderate	Wheat; barley; sugar beet; maize	Almonds, grapes, pomegranates, damask rose; figs
Yazd	Yazd	31.90	54.28	1237.2	Extra arid—Cold	Sorghum, fodder maize, millet, legumes, alfalfa	Pistachios, pomegranates, apricots, saffron

The data used in this paper include monthly meteorological data that belong to the period of 1995 to 2018. These data include minimum air temperature (Tmin), maximum air temperature (Tmax), mean air temperature (Tmean), minimum relative humidity (RHmin), maximum relative humidity (RHmax), and sunshine duration (SSD), which are prepared on a monthly scale of the Iranian Meteorological Organization (IRIMO). The temperature and humidity variables (Tmin, Tmax, Tmean, RHmax and RHmin) are measured in Stevenson screen box at 1.35 m height from the land surface, and the SSD is measured by sunshine recorder at 1.5 m height. The quality control of these datasets have been checked. There were a few numbers of missings and outliers that were modified using averaging method. Using these data and the FAO-56 PM model, the amount of monthly evapotranspiration was calculated in the six mentioned stations. The reference evapotranspiration based on the FAO-56 PM method is calculated by Eq. (1):

1 $$ET0 = { }\frac{{0.408\Delta \left( {R_{n} - G} \right) + \gamma \frac{900}{{(T_{a} + 273)}}u_{2} \left( {e_{s} - e_{a} } \right)}}{{\Delta + \gamma \left( {1 + 0.34u_{2} } \right)}}$$

where $ET0,{ }\Delta ,{ }R_{n} ,{ }G,{ }\gamma ,{ }T_{a} ,{ }u_{2} ,\;{\text{and}}\;e_{s} - { }e_{a} { }$ represent refrence evapotranspirartion $\left( {\frac{{{\text{mm}}}}{{{\text{month}}}}} \right)$, the slope of the vapor pressure curve $\left( {\frac{{{\text{kP}}_{{\text{a}}} }}{{^\circ {\text{C}}}}} \right)$, net surface radiation $\left( {\frac{{{\text{MJ}}}}{{{\text{m}}^{2} {\text{day}}}}} \right)$, soil heat flux ($\left( {\frac{{{\text{MJ}}}}{{{\text{m}}^{2} {\text{day}}}}} \right)$, psychrometric constant $\left( {0.0677\frac{{{\text{ kP}}_{{\text{a}}} }}{{^\circ {\text{C}}}}} \right)$, monthly mean air temperature ($^\circ {\text{C}}$), monthly average wind speed $\left( {\frac{{\text{m}}}{{\text{s}}}} \right)$ at 2 m, and the difference between saturation and actual vapor pressure ($kP_{a}$), respectively (Allen et al.^1^). According to Allen et al.^1^, $\Delta ,{ }\gamma$ were computed as a function of atmospheric pressure obtained from the local altitude (m), and the maximum and minimum relative humidity as well as maximum and minimum temperature values were used to compute $e_{a}$ and $e_{s}$. Net radiation is the difference between net incoming shortwave solar radiation and outgoing longwave terrestrial radiation. Due to the lack of measurement of the actual solar radiation in most synoptic stations like the one in this research, solar radiation can be estimated from the Angstrom formula based on the actual sunshine duration. Moreover, the net output longwave radiation was estimated according to the modified Stefan-Boltzmann law by considering the effect of cloudiness and atmospheric humidity (downward longwave from the sky). Interested readers can refer to Allen et al. ^1^. The “evapotranspiration” package in R software was used to estimate the evapotranspiration rates. For modeling, the period under study was divided into two parts of training and testing that include 75% (the first 18 years of 1995–2012) and 25% (the remaining six years of 2013–2018), respectively. In the training phase, the model is extracted, and the extracted model is applied for predicting ET0 during the testing phase. Then the models’ predictions will be validated by the actual (calculated) ET0. The characteristics of the meteorological data and the calculated evapotranspiration data are shown in Table 2.

**Table 2:** Specifications of the meteorological data used and the calculated ET0 on the monthly scale.

Station	Variable	Training period (1995–2012)				Testing Period (2013–2018)			
		Min.*	Max	Average	STD	Min	Max	Average	STD
Bandar Anzali	Tmin (°C)	0.80	25.40	14.41	6.85	3.10	26.10	14.80	6.84
	Tmax (°C)	5.30	31.80	19.24	7.14	8.40	32.80	20.12	7.57
	Tmean (°C)	3.00	28.40	16.82	6.99	5.80	29.30	17.46	7.18
	RHmax (%)	81.20	96.90	92.21	3.09	81.50	96.50	91.68	3.73
	RHmin (%)	54.80	84.10	73.11	5.72	53.90	84.40	71.76	7.04
	SSD ($\frac{{{\text{hr}}}}{{{\text{month}}}}$)	28.50	337.60	161.74	73.68	40.40	339.70	163.78	82.92
	ET0 ($\frac{{{\text{mm}}}}{{{\text{month}}}}$)	20.60	174.30	74.39	43.57	22.70	170.30	80.42	49.65
Ramsar	Tmin (°C)	0.90	24.90	13.77	6.82	2.90	25.40	14.34	6.85
	Tmax (°C)	7.10	31.50	19.93	6.86	9.20	32.50	20.43	7.23
	Tmean (°C)	4.00	28.20	16.86	6.82	6.10	28.90	17.39	7.03
	RHmax (%)	80.60	97.30	89.85	3.33	80.30	95.10	90.18	3.80
	RHmin (%)	56.50	84.20	69.07	4.83	56.70	82.70	69.61	5.82
	SSD ($\frac{{{\text{hr}}}}{{{\text{month}}}}$)	39.00	289.20	139.53**	51.16	52.80	309.70	140.29	58.79
	ET0 ($\frac{{{\text{mm}}}}{{{\text{month}}}}$)	20.90	158.50	**71.52**	37.90	23.20	151.70	72.77	42.10
Gharakhil	Tmin (°C)	-1.30	23.80	12.76	7.14	1.50	24.20	13.03	7.20
	Tmax (°C)	8.10	34.80	21.98	7.14	11.70	34.70	22.58	7.35
	Tmean (°C)	3.40	28.80	17.37	7.11	6.60	29.20	17.80	7.26
	RHmax (%)	89.40	98.90	**95.40**	2.04	89.20	97.00	94.16	2.07
	RHmin (%)	46.50	76.90	62.45	5.59	47.60	73.50	62.27	5.41
	SSD ($\frac{{{\text{hr}}}}{{{\text{month}}}}$)	40.30	310.20	170.11	49.43	73.30	317.60	169.54	53.09
	ET0 ($\frac{{{\text{mm}}}}{{{\text{month}}}}$)	23.40	164.40	78.10	40.16	20.20	169.70	80.22	44.70
Ahwaz	Tmin (°C)	6.20	31.50	19.44	7.86	7.40	31.40	19.79	8.02
	Tmax (°C)	14.70	48.10	**33.60**	10.59	17.40	48.90	34.15	10.24
	Tmean (°C)	10.40	39.80	26.52	9.20	13.40	39.90	26.98	9.10
	RHmax (%)	28.10	95.80	60.09	19.00	27.80	96.30	62.35	18.27
	RHmin (%)	6.80	67.10	23.85	14.67	7.80	64.70	25.46	13.46
	SSD ($\frac{{{\text{hr}}}}{{{\text{month}}}}$)	162.40	383.60	273.79	58.02	163.60	370.30	272.99	58.36
	ET0 ($\frac{{{\text{mm}}}}{{{\text{month}}}}$)	40.20	354.50	**169.06**	93.21	44.80	310.50	161.89	85.55
Shiraz	Tmin (°C)	-2.00	24.20	**10.95**	7.46	-1.10	22.30	10.46	7.29
	Tmax (°C)	9.40	40.10	26.33	9.17	11.70	40.10	26.90	8.85
	Tmean (°C)	4.80	32.10	18.64	8.26	5.60	31.10	18.68	8.04
	RHmax (%)	30.00	91.90	58.33	17.96	27.80	90.90	58.51	18.24
	RHmin (%)	6.60	54.50	20.86	11.01	4.30	49.50	17.51	10.04
	SSD ($\frac{{{\text{hr}}}}{{{\text{month}}}}$)	208.50	372.30	**296.88**	40.68	222.70	370.30	294.97	40.10
	ET0 ($\frac{{{\text{mm}}}}{{{\text{month}}}}$)	37.90	251.40	133.79	64.01	44.70	224.50	129.44	60.15
Yazd	Tmin (°C)	-4.40	28.30	13.24	8.74	1.10	27.40	14.32	8.46
	Tmax (°C)	4.80	42.60	27.33	9.62	12.40	41.80	27.87	9.05
	Tmean (°C)	0.20	35.50	20.29	9.16	6.80	34.60	21.10	8.74
	RHmax (%)	15.50	87.70	41.06	19.22	12.60	80.40	38.11	17.38
	RHmin (%)	5.10	57.60	**16.25**	9.96	4.90	39.60	14.49	7.54
	SSD ($\frac{{{\text{hr}}}}{{{\text{month}}}}$)	209.80	376.80	292.77	47.08	200.40	383.00	296.97	47.65
	ET0 ($\frac{{{\text{mm}}}}{{{\text{month}}}}$)	34.00	289.10	156.13	73.86	55.30	273.50	155.87	70.35

*Min. = Minimum; Max. = Maximum; STD = Standard deviation.

**The rows bolded in this comment, show the extreme values of the variable. For example, the minimum values of Tmin and RHmin, belong to the Shiraz and Yazd stations, respectively. Or the maximum values of Tmax and RHmax, belong to the Ahwaz and Gharakhil stations, respectively. For the variables SSD and ET0, both minimum (Ramsar) and maximum values (Shiraz and Ahwaz) are bolded.

### Time series model

A time series is a set of recorded observations of a variable such as ${\text{X}}_{{\text{i}}}$ Overtime in the form of ${\text{X}}_{1}$, ${\text{X}}_{2}$, ${\text{X}}_{3}$, …, ${\text{X}}_{{\text{N}}} ,$ among which the time interval is equal (Gautam and Sinha^54^). Time series models are stochastic models that work based on regression coefficients and use the time lags of the target variable as the model’s input variable. These models include autoregressive (AR), integrated (I), and moving average (MA) components. They are shown in an integrated state known as autoregressive integral moving average (ARIMA). The seasonal ARIMA (SARIMA) model is a model that can be used for numerical simulation of the stochastic behavior of periodic time series. In other words, SARIMA is a linear parametric stochastic model that can be used to model and predict variables which have seasonal autocorrelations. The cross form of this model is shown as SARIMA(p, d, q) × (P, D, Q)_ω_, in which ω is the periodicity, p, d, and q are the non-seasonal degrees of autoregressive, differencing, and moving average, respectively, and P, D, and Q are the seasonal degrees of autoregressive, differencing, and moving average, respectively. The general form of this model is shown below: (Salas^55^):

2 $$\Phi_{P} \left( {B^{\omega } } \right)\phi_{p} \left( B \right)\nabla_{\omega }^{D} \nabla^{d} X_{t} = \theta_{q} \left( B \right)\Theta_{Q} \left( {B^{\omega } } \right)\varepsilon_{t}$$

In this formula ${X}_{t}$ is a stochastic variable as the target, and ${\varepsilon }_{t}$ is a normal random variable with mean μ and variance ${\sigma }_{\varepsilon }^{2}$, as a residual. The parameters of B including Φ, ϕ, ${\nabla }_{\omega }^{D}$, ${\nabla }^{d}$, Θ, θ, represent the backward operators associated with seasonal autoregressive, non-seasonal autoregressive, seasonal differencing and non-seasonal differencing, seasonal moving average, and non-seasonal moving average, respectively. Their equations are described in Eqs. 3–8 (Salas^55^).

3 $$\Phi_{P} \left( {B^{\omega } } \right) = \left( {1 - \Phi_{1} B^{\omega \times 1} - \ldots - \Phi_{P} B^{\omega \times P} } \right)$$

4 $$\phi_{p} \left( B \right) = \left( {1 - \phi_{1} B^{1} - \ldots - \phi_{p} B^{p} } \right)$$

5 $$\nabla_{\omega }^{D} = \left( {1 - B^{\omega } } \right)^{D}$$

6 $$\nabla^{d} = \left( {1 - B} \right)^{d}$$

7 $$\Theta_{Q} \left( {B^{\omega } } \right) = \left( {1 - \Theta_{1} B^{\omega \times 1} - \ldots - \Theta_{Q} B^{\omega \times Q} } \right)$$

8 $$\theta_{q} \left( B \right) = \left( {1 - \theta_{1} B^{1} - \ldots - \theta_{q} B^{q} } \right)$$

We used the Minitab software and the SARIMA model to simulate and predict evapotranspiration time series in this research.

### Adaptive neuro-fuzzy inference system (ANFIS)

**Figure 2:**
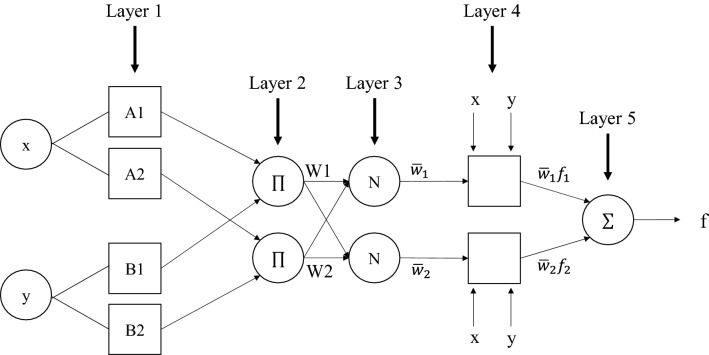
The schematic structure of an ANFIS model with two inputs.

ANFIS model can make relationships between input and output data using fuzzy rules to learn from a neural network to generate input structure for a system. ANFIS model designs and creates nonlinear maps to define relationships between input and output spaces by employing the artificial neural network and fuzzy logic, which is known as a neuro-fuzzy system. fuzzy systems include three different parts: fuzzification, inference engine, and defuzzification. By utilizing fuzzy inference systems, fuzzy rules are achieved. A fuzzy inference system consists of two different inferences, namely Mamdani (Mamdani and Assilian^56^) and Sugeno (Takagi and Sugeno^57^). They both work great when combined with an optimization algorithm and adaptive techniques (Khosravi et al.^39^). In this paper, we use Sugeno inference. Figure 2 shows the structure of the ANFIS model.

These two equations are the base rules of Sugeno inference:

9 $${\text{Rule 1}}:\;{\text{if}}\; x\; is \;A_{1} \;{\text{and}}\; y \;is\; B_{1} , \;{\text{then}}\; f_{1} = p_{1} x + q_{1} y + r_{1}$$

10 $${\text{Rule 1}}:\;{\text{if}}\; x\; is \;A_{2} \;{\text{and}} \;y \;{\text{is}} \;B_{2} , \;{\text{then}}\; f_{2} = p_{2} x + q_{2} y + r_{2}$$

ANFIS model contains different layers. Layer one, in this model, is the fuzzification layer. Each node receives a signal and then transfers it to the next layer. The following equation describes the cells’ outputs ($O_{1}^{i}$) (Khosravi et al.^39^; Haznedar and Kalinli^58^):

11 $$O_{1}^{i} = \mu_{{A_{i} }} \left( x \right);\quad i = 1, 2$$

${\mu }_{{A}_{i}}$ is related to membership function (MF). ${A}_{i}$ is linguistic variable and is related to node function. The following equation shows the standard formula for ${\mu }_{{A}_{i}}$

12 $$\mu_{{A_{i} }} \left( x \right) = \exp \left\{ { - \left[ {\left( {\frac{{x - c_{i} }}{{a_{i} }}} \right)^{2} } \right]^{{b_{i} }} } \right\}$$

In this equation, *x* is the input, and ${a}_{i}$, ${b}_{i}$, ${c}_{i}$ are premise parameters. Layer 2 is called the rule layer which is obtained by membership degrees. All the output nodes establish the firing strength of a fuzzy rule.

13 $$O_{2}^{i} = w_{i} = \mu_{{A_{i} }} \left( x \right){ } \cdot \mu_{{B_{i} }} \left( y \right);\quad i = 1, 2$$

Layer 3 is the normalization layer. In this layer, all the nodes are fixed and tagged with N. The rule’s firing strength to the sum of all rules’ firing strengths is the ratio calculated by the $i^{th}$ node in the normalization layer.

14 $$O_{3}^{i} = \overline{{w_{i} }} = \frac{{w_{i} }}{{w_{1} + w_{2} }};\quad i = 1, 2$$

The defuzzification layer is layer 4 of the ANFIS model. Each rule uses the value of the previous layer to compute the output value.

15 $$O_{4}^{i} = \overline{{w_{i} }} f_{i} = \overline{{w_{i} }} \left( {p_{i} x + q_{i} y + r_{i} } \right);\quad i = 1, 2$$

In this equation, $\overline{{w_{i} }}$ comes from the previous layer, namely layer 3. $\overline{{w_{i} }}$ is a normalized firing strength and $p_{i}$, $q_{i}$, and $r_{i}$ are the consequent parameters. Layer 5 is called the sum layer. By summing the output values of the rules that come from the previous layer, the final output of the ANFIS model is calculated.

16 $$O_{5}^{i} = overall \; output = \mathop \sum \limits_{i} \overline{{w_{i} }} f_{i} = \frac{{\mathop \sum \nolimits_{i} w_{i} f_{i} }}{{\mathop \sum \nolimits_{i} w_{i} }}\quad i = 1, 2$$

To implement the ANFIS model, we used MATLAB software in this study.

To summarize, the ANFIS model contains two sets of parameters: premise parameters and consequence parameters. Premise parameters are input parameters of MFs, and they aim to specify the shape and the location of the input MFs (parameters of input MFs). Consequence parameters are the output parameters of MFs (parameters of output MFs) (Jang ^59^). Classical ANFIS uses the least square (LS) methods to estimate these parameters. However, in the current research, we have developed a novel ANFIS-DE model, which uses the meta-heuristic DE algorithm to estimate ANFIS’s sets of parameters.

### Differential evolution (DE) optimization algorithm

Although differential evolution (DE) uses basic optimized operations such as mutation, crossover, and selection, it is an impressive and powerful optimization algorithm. One of the privileges of this algorithm is that it has parallel search methods and uses NP, and also has D-dimensional vectors of parameters (Omidi and Mazaheri^60^). The advantage of these vectors is that they do not change during the minimization procedure. DE performs a population process for each generation G. First, one population vector is randomly initialized, including the parameters, and this probability distribution is uniformed. When the preliminary solution is achieved, the DE algorithm calculates the difference between the weights of two population vectors and assigns it to the third vector in order to produce new parameter vectors, which is known as the mutation operation (Halabi et al.^34^):

17 $$v_{i,G + 1} = x_{i,G} + F\left( {x_{r2,G} - x_{r3,G} } \right)$$

According to $v_{i,G + 1}$, these mutant vectors, $x_{i}$, $G$ and $i = 1,2,3, \ldots ,NP$ are created, while $r1$, $r2$, and $r3$ are randomly integers, and NP is selected from this distribution: integers $\in \left[ {1,2,3, \ldots ,NP} \right].$ Moreover, $I$ and $F$ are real values, and they are different $\in \left[ {1,2,3, \ldots ,NP} \right]$.

During the mixing process, which is also called crossover operation, parameters of the mutated vector are mixed with other vector parameters to create the trial vector. The following equations describe this mixing process:

18 $$u_{i,G + 1} = \left( {u_{1i,G + 1} ,u_{2i,G + 1} , \ldots ,u_{di,G + 1} } \right)$$

19 $$u_{ji,G + 1} = \left\{ {\begin{array}{*{20}c} {v_{ji,G + 1} ; if \, randb\left( j \right) \le \; CR \; or \; j = rnbr\left( i \right)} \\ {x_{ji,G + 1} ; if \; randb\left( j \right) > \; CR \; or \; j \ne rnbr\left( i \right)} \\ \end{array} } \right.$$

**Figure 3:**
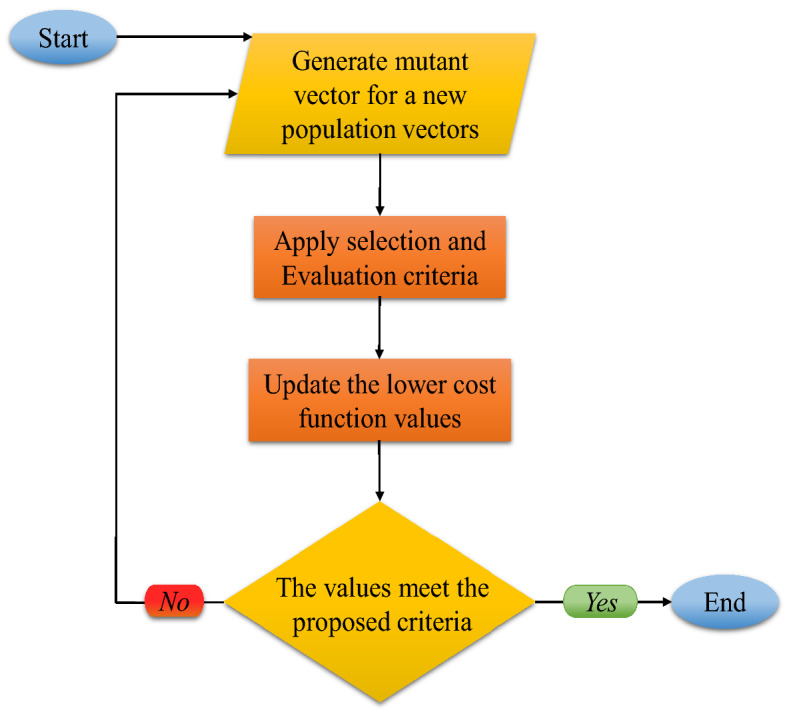
Flowchart of the optimization process based on differential evolution algorithm.

In this equation, $u_{i,G + 1}$ is the trailer, and $x_{i,G}$ is the target vector, where $u_{i,G + 1}$ and $x_{i,G}$ are the trailer and target vectors, respectively. $randb\left( j \right)$ is the *J*th uniform random evaluation $\in \left[ {0.1} \right]$, $rnbr\left( i \right)$ is a random value index $\in \left[ {1,2,3, \ldots ,d} \right],$ and $CR$ is a crossover constant determined by users. The selection operation is the last step. The trial vector costs a lower cost function than the target vector. Therefore, the selection operation uses the trial vector as a target value for the next generation. $NP$ competitions are assumed like one generation procedure as each population vector has to serve once as the target vector. Complementary descriptions about the DE optimization algorithm can be found in Storn and Price^61^ and Halabi et al.^34^. The DE algorithm flowchart is illustrated in Fig. 3.

In this paper, the DE algorithm is implemented by coding in MATLAB software’s environment. The trial and error method is used to choose the best operators of DE to optimize the ANFIS model. They are illustrated in Table 3.

**Table 3:** The operators of differential evolution algorithm.

Operator	Value
Population	100
Maximum number of iterations	200
Crossover probability	0.1
Scaling factor lower bound	0.2
Scaling factor upper bound	0.8

### Evaluating the accuracy of the predictions

This study uses six criteria to evaluate the performance of the models: root mean square error (RMSE), normalized RMSE (NRMSE), mean absolute error (MAE), percent bias (PB), Pearson correlation coefficient (*R*), coefficient of determination (*R*^2^), and Nash- Sutcliff coefficient (NS). In general, these criteria are used to compare the accuracy of different models with one another. Furthermore, they are used to compare the accuracy of models in different climates. To calculate them, we need two series of predicted and observed evapotranspiration data. Their equations are as follows.

20 $$RMSE = \sqrt {\frac{1}{n}\mathop \sum \limits_{i = 1}^{n} \left( {ETO_{i} - ETP_{i} } \right)^{2} } ;\quad 0 < RMSE < + \infty$$

21 $$NRMSE = \frac{{\sqrt {\frac{1}{n}\mathop \sum \nolimits_{i = 1}^{n} \left( {ETO_{i} - ETP_{i} } \right)^{2} } }}{{ETO_{max} - ETO_{min} }};\quad 0 < NRMSE < + \infty$$

22 $$MAE = \frac{1}{n}\mathop \sum \limits_{i = 1}^{n} \left| {ETO_{i} - ETP_{i} } \right|;\quad 0 < MAE < + \infty$$

23 $$PB = \mathop \sum \limits_{i = 1}^{n} \left( {\frac{{ETO_{i} - ETP_{i} }}{{ETO_{i} }}} \right);\quad - \infty < PB < + \infty$$

24 $$R = \frac{{\mathop \sum \nolimits_{i = 1}^{n} \left( {ETO_{i} - \overline{ETO} } \right)\left( {ETP_{i} - \overline{ETP} } \right)}}{{\sqrt {\mathop \sum \nolimits_{i = 1}^{n} \left( {ETO_{i} - \overline{ETO} } \right)^{2} } *\sqrt {\mathop \sum \nolimits_{i = 1}^{n} \left( {ETP_{i} - \overline{ETP} } \right)^{2} } }};\quad - 1 < R < 1$$

25 $$R^{2} = \left[ {\frac{{\mathop \sum \nolimits_{i = 1}^{n} \left( {ETO_{i} - \overline{ETO} } \right)\left( {ETP_{i} - \overline{ETP} } \right)}}{{\sqrt {\mathop \sum \nolimits_{i = 1}^{n} \left( {ETO_{i} - \overline{ETO} } \right)^{2} } *\sqrt {\mathop \sum \nolimits_{i = 1}^{n} \left( {ETP_{i} - \overline{ETP} } \right)^{2} } }}} \right]^{2} ;\quad 0 < R^{2} < 1$$

26 $$NS = 1 - \frac{{\mathop \sum \nolimits_{i = 1}^{n} \left( {ETO_{i} - ETP_{i} } \right)^{2} }}{{\mathop \sum \nolimits_{i = 1}^{n} \left( {ETO_{i} - \overline{ETO} } \right)^{2} }};\quad - \infty < NS < 1$$

$ETO_{i }$ shows the amount of the observed evapotranspiration (FAO-56 PM calculated ET0) of the *i*th month, $ETP_{i}$ is the amount of evapotranspiration predicted in the *i*th month, $\overline{ETO}$ shows the mean of observed evapotranspiration, $\overline{ETP}$ represents the average of the predictive evapotranspiration, $ETO_{max}$ is the maximum of the observed evapotranspiration, and finally $ETO_{min}$ is the minimum of the observed evapotranspiration. According to the defined range for these criteria, the closer the RMSE, NRMSE, MAE and PB are to zero, and the closer NS, *R*, and *R*^2^ are to one, the better the model performance is. Another point about NRMSE is that it has 4 intervals while evaluating the models’ quality: (1) NRMSE > 0.3 poor performance, (2) 0.2 < NRMSE < 0.3 average performance (3) 0.1 < NRMSE < 0.2 good performance and (4) 0 < NRMSE < 0.1 excellent performance (Bahrami-Pichaghchi and Aghelpour^62^). From another point of view, the used criteria are divided into four categories: (I) Accuracy: these criteria can show the errors of the models in ET0 prediction, including RMSE, MAE; (II) precision: these criteria can show the quality of the models in ET0 prediction, including NRMSE and NS; (III) under or overestimation: this criterion can talk about the models’ under/overestimation in ET0 prediction, including PB; (IV) correlation: these criteria show the correlation intensity between the models’ predictions and their observed values, including *R* and *R*^2^. It should be noted that all these criteria will be applicable for comparing several models in a specific station.

**Figure 4:**
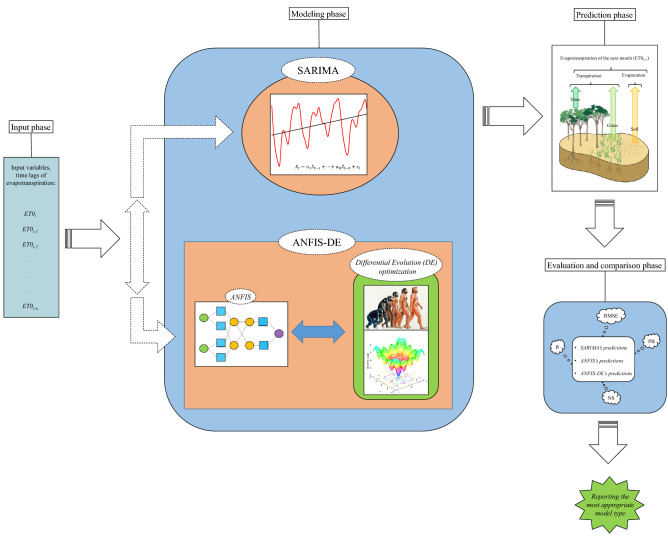
General flowchart of the evapotranspiration modeling, prediction, and evaluation processes.

The general process of modeling and predicting the evapotranspiration time series in this paper is shown as a flowchart in Fig. 4.

## Results

### Modeling and evaluating the predictions

**Figure 5:**
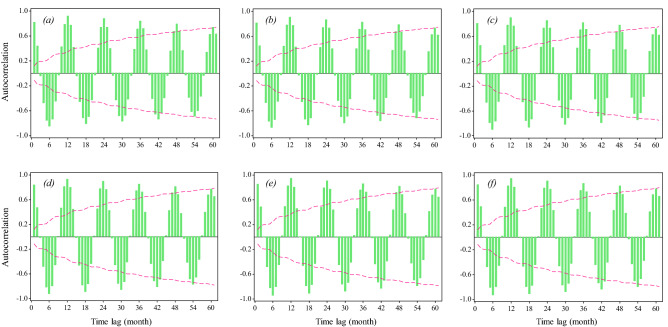
Autocorrelation plots for the monthly ET0 time series; the alphabets within the brackets refer to the stations: (**a**) Bandar Anzali, (**b**) Ramsar, (**c**) Gharakhil, (**d**) Ahwaz, (**e**) Shiraz, (**f**) Yazd.

In this study, the ET0 rates were first calculated by FAO-56 PM method, and the meteorological variables, are represented in Table 1. Then the models were applied for ET0 prediction. It’s worth mentioning that if the inputs are the meteorological variables, the modeling problem is applicable for an “estimation” case and is not usable for a “prediction” (for the future). For a time series prediction problem, the model inputs must have time lag(s) and the output’s time lag must be equal to zero. Since the time series stochastic models are only able to consider the main variable's time lags as input, the same inputs (time lags of ET0) are considered for the ANFIS and ANFIS-DE models too, for a fair comparison. Therefore, autocorrelation function (ACF) diagrams for different stations were considered (Fig. 5) that show the extent and significance of the correlation of the variable with its previous steps’ amounts.

As Fig. 5 indicates, the ET0 data in all six stations have a significant seasonal trend. The ET0 time series are periodic and have a 12 months periodicity. To moderate this seasonal trend, we considered several degrees of seasonal differentiations with a lag of 12 months (equal to the periodicity). Investigations showed that order “one” seasonal differentiation has the best consistency with ET0 data. As a result, the SARIMA model is modified as the SARIMA pattern SARIMA (p,0,q)(P,1,Q)_12_. Moreover, when the time lag increases, the significance threshold of correlation (dashed line) increases; with more than three return periods (36 months), it reaches a point that is practically logical not to use them as inputs. Therefore, a maximum lag of 36 months is considered as input for all models. In the SARIMA model, this includes seasonal autoregressive and moving average degrees (P & Q), which are equal to 1, 2, and 3. These degrees and also the non-seasonal degrees of autoregressive and moving average (p & q) were all tested, and their best performance was selected for each station and reported in Table 4. Simple and hybrid ANFIS models (ANFIS & ANFIS-DE) were implemented based on the fuzzy cluster means (FCM) clustering method. Lags of 1, 6, 12, 18, 24, 30, and 36 months were also considered as inputs to these AI models.

**Table 4:** Evaluating the models’ predictions by evaluation criteria.

Station	Model	Train			Test		
		RMSE ($\frac{{{\text{mm}}}}{{{\text{month}}}}$)	PB	*R*	RMSE ($\frac{{{\text{mm}}}}{{{\text{month}}}}$)	PB	*R*
Bandar Anzali	**SARIMA(1,0,0)(2,1,2)**_12_*	**9.436**	− **0.026**	**0.977**	**10.078**	**− 0.042**	**0.982**
	ANFIS	8.177	− 0.014	0.983	12.767	0.035	0.970
	ANFIS-DE	10.492	− 0.019	0.971	10.532	− 0.018	0.977
Ramsar	**SARIMA(1,0,2)(3,1,3)**_**12**_	**8.973**	− **0.011**	**0.973**	**9.711**	**− 0.028**	**0.975**
	ANFIS	8.130	− 0.011	0.977	13.257	− 0.013	0.949
	ANFIS-DE	11.171	− 0.015	0.957	10.998	− 0.013	0.965
Gharakhil	**SARIMA(1,0,0)(3,1,1)**_**12**_	**10.909**	− **0.013**	**0.963**	**9.713**	**− 0.041**	**0.979**
	ANFIS	9.624	− 0.014	0.971	12.569	− 0.018	0.960
	ANFIS-DE	12.300	− 0.018	0.953	10.711	− 0.005	0.970
Ahwaz	**SARIMA(1,0,1)(2,1,3)**_**12**_	**14.844**	**− 0.003**	**0.987**	**12.789**	**0.020**	**0.990**
	ANFIS	12.597	− 0.008	0.991	16.906	− 0.021	0.983
	ANFIS-DE	16.134	− 0.008	0.984	14.533	− 0.020	0.985
Shiraz	**SARIMA(1,0,1)(2,1,2)**_**12**_	**8.364**	**− 0.004**	**0.991**	**7.918**	**0.013**	**0.992**
	ANFIS	6.281	− 0.004	0.995	9.920	− 0.007	0.986
	ANFIS-DE	10.408	− 0.009	0.987	9.077	− 0.014	0.988
Yazd	**SARIMA(2,0,0)(3,1,3)**_**12**_	**10.142**	**− 0.007**	**0.991**	**8.897**	**0.005**	**0.994**
	ANFIS	8.858	− 0.008	0.993	10.537	0.007	0.989
	ANFIS-DE	11.224	− 0.011	0.989	9.548	0.000	0.991

*Bold rows specify the best-fitted model in each station.

In Table 4, the predictions of all three models were evaluated by the mentioned evaluation metrics. Since the test section actually shows the validity of the models, the test section is also discussed in the interpretations of this section. At first, it can be seen that in all stations, the R coefficients are very high, which indicates the optimal performance of the models in predicting monthly ET0 (the minimum value of R is equal to 0.949, which belongs to the simple ANFIS model in Ramsar station). Additionally, the amount of PB in all cases is very small (close to zero), which confirms the lack of significant under/overestimation and, consequently, the excellent performance of the models. According to Table 4, the SARIMA linear model has superior performance in all stations than the other two models, and the weakest performance among the models belongs to the simple ANFIS model. In combination with the ANFIS model (ANFIS-DE), the DE algorithm was able to increase the prediction accuracy for ANFIS by an average of 15.8%. The lowest prediction error belongs to the SARIMA model at Shiraz station with RMSE = 7.918 $\frac{{{\text{mm}}}}{{{\text{month}}}}$. The highest prediction error is reported in Ahwaz station with RMSE = 16.906 $\frac{{{\text{mm}}}}{{{\text{month}}}}$ , which belongs to the simple ANFIS model.

### Comparing the models

**Figure 6:**
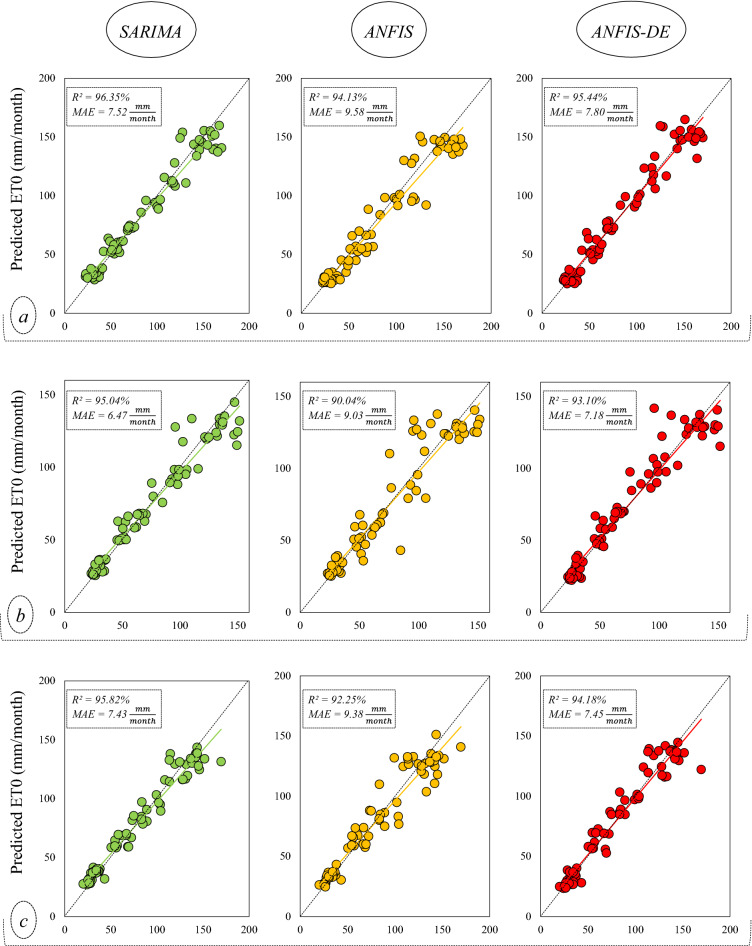

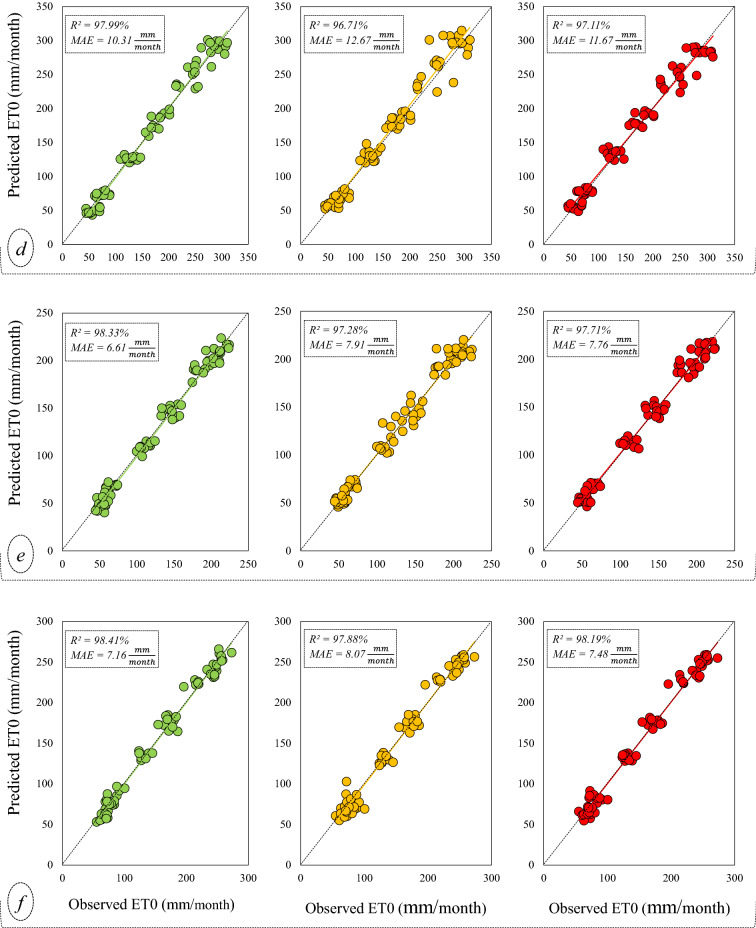
Scatter plots to investigate the models’ predictions against their simultaneous observed values; the alphabets within the brackets refer to the stations: (**a**) Bandar Anzali, (**b**) Ramsar, (**c**) Gharakhil, (**d**) Ahwaz, (**e**) Shiraz, (**f**) Yazd.

Scatter plots are used for graphical illustration of the correlation between the predicted and actual values of monthly ET0 (Fig. 6).

**Figure 7:**
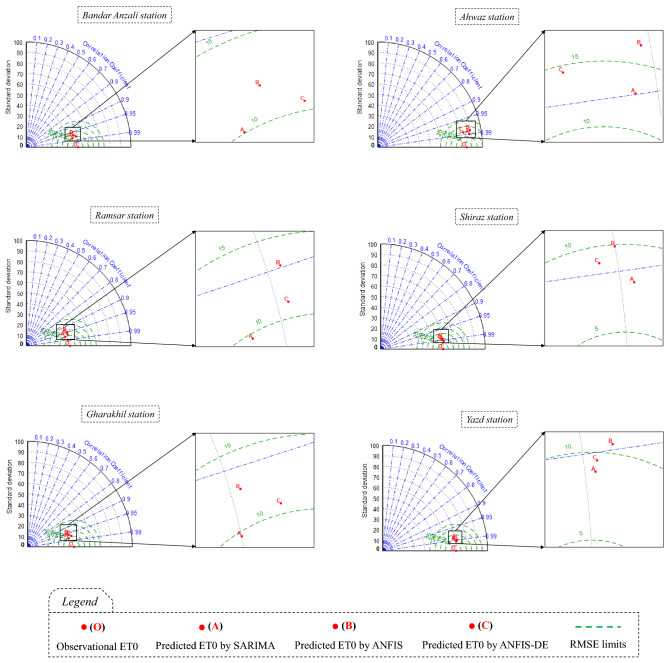
Taylor diagrams to compare the models in the stations; the diagram of each station is specified by its own name.

In Fig. 6, the horizontal axis of the graphs represents the observed ET0 data, and the vertical axis represents the predictions presented by the models. This figure shows that, at all stations, the slope of the fitted regression line between the observed-predicted data samples is very small, associated with the X = Y line. The points are well concentrated around their regression line, and this concentration is more on the diagrams related to the SARIMA model than the other two models. On the other hand, the *R*^2^ coefficient shows that the SARIMA linear model offers a better prediction than the other two nonlinear and complex models, i.e., ANFIS and ANFIS-DE. Also, ANFIS-DE predictions show better correlations compared to simple ANFIS. The diagrams in Fig. 6 show that the weakest performance belongs to the predictions of ANFIS in Ramsar (*R*^2^ = 0.901), and the best performance belongs to the predictions of SARIMA at Yazd station (*R*^2^ = 0.984). The Taylor diagram is also represented for each station to compare the models (Fig. 7).

This diagram (Fig. 7) can simultaneously check the correlation and the error and also compare the standard deviations of the outputs of several models and their observed values. In these diagrams, point O is an indicator of observed data, and points A, B, and C are the indicators of the SARIMA, ANFIS, and ANFIS-DE models, respectively. At all stations, point A is located closest to point O, confirming the superiority of the SARIMA model. After that, ANFIS-DE (point C) and ANFIS (point B) models are located in the second and third places, respectively. The best position of points A, B, and C belongs to Shiraz station, where these points are placed between two circles RMSE = 5 $\frac{{{\text{mm}}}}{{{\text{month}}}}{ }$ and RMSE = 10 $\frac{{{\text{mm}}}}{{{\text{month}}}}$, and around the radius *R* = 0.99. At Yazd station, a situation similar to Shiraz station is observed. The weakest points’ position can belong to Bandar Anzali station, where points A, B, and C are farthest from point O, between circles of RMSE = 10 $\frac{{{\text{mm}}}}{{{\text{month}}}}{ }$ and RMSE = 15 $\frac{{{\text{mm}}}}{{{\text{month}}}}$, and between two radii of *R* = 0.99 and *R* = 0.95. Furthermore, a comparison of the standard deviations between outputs and the observations reveals that the points of the models, especially point A, are in a favorable position relative to the quadrant close to point O. This shows that the models, especially SARIMA, can favorably estimate the standard deviation of actual ET0 values.

### Comparing ET0 prediction accuracy in different climates

**Figure 8:**
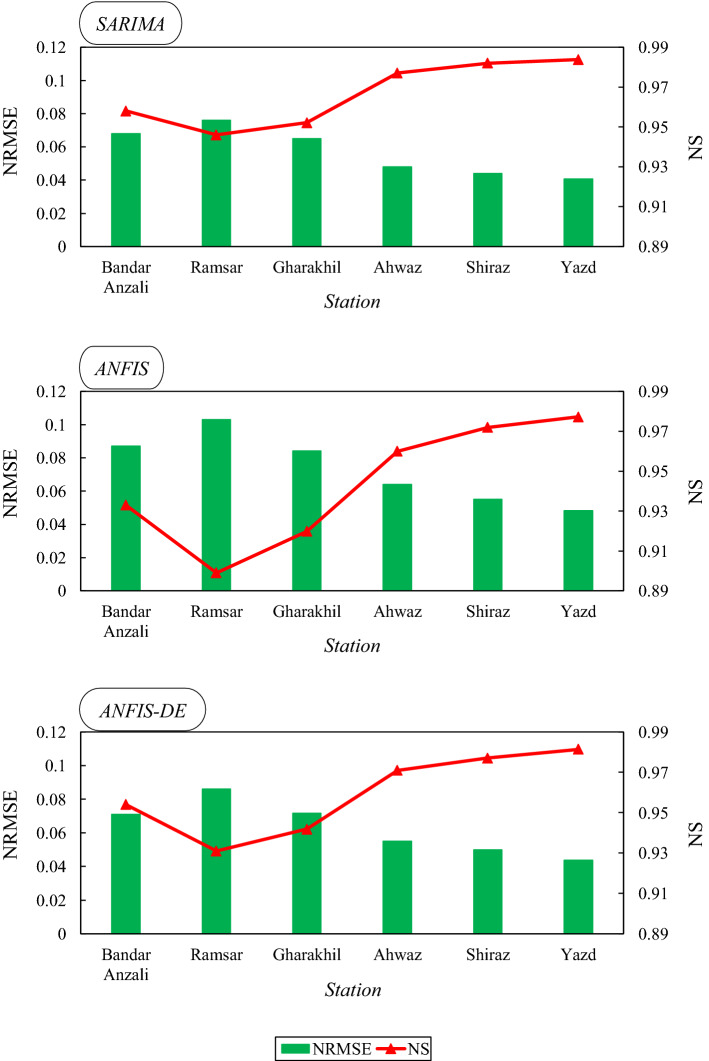
Combo-graph of NRMSE and NS criteria to make a comparison between the different climates.

In general, the comparison between the stations in Fig. 7 indicates that the humid stations are in weaker ranges of error and correlation than the arid stations. Also, according to Fig. 6, the R^2^ value resulting from the SARIMA model in humid and sub-humid climates is in the range of 0.95–0.96, while it is in the range of 0.97–0.98 in arid and semi-arid regions. Therefore, it is evident that ET0 is predicted slightly better in arid areas. However, due to the different range of ET0 data in different climates (Table 2), it is better to consider the normalized RMSE (NRMSE) criterion at stations for evaluation (Fig. 8).

**Figure 9:**
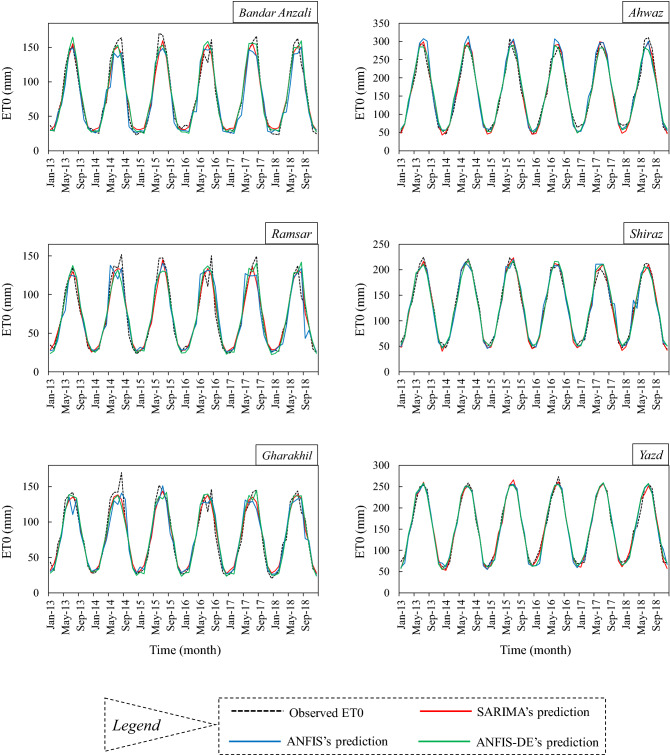
Multiple time series plots of the observed monthly evapotranspiration beside the models’ predictions.

In Fig. 8, the NRMSE and NS criteria for the test period were plotted together as a combo-graph. This diagram is drawn separately for all models at all stations. At first, we can observe that all models have an NS value greater than 0.9, which confirms the models’ favorable prediction of ET0. Moreover, the NRMSE value in all stations is less than 0.1. According to the quality classes defined for NRMSE, the predictions for all climates in this study are considered very reasonable. The visible trend of NS and NRMSE is similar across stations. Both criteria indicate a better prediction of ET0 in arid and semi-arid climates. In other words, if the NS level increases at a station, the NRMSE level will decrease at the same station (which is well illustrated in the combo-graph). Therefore, we can state that both criteria achieved similar results in comparing the accuracy of ET0 prediction among the climates. For example, in the ANFIS-DE model for humid and sub-humid stations, the NRMSE is between 0.07 and 0.09 and the NS is between 0.93 and 0.95, while for arid and semi-arid stations, NRMSE is between 0.04 and 0.06 and NS is between 0.97 and 0.98. In the combo-graph belonging to the SARIMA model, the NRMSE value for humid and sub-humid areas is between 0.06 and 0.08, and the NS value is between 0.94 and 0.96, while for arid and semi-arid areas, the NRMSE is between 0.04 and 0.05, and the NS is between 0.98 and 0.99. The comparison of the models is similar to the previous diagrams and tables, which reported that the SARIMA model is more appropriate. The predictions provided by the models can also be seen graphically in time-series plots (Fig. 9) to observe the overlaps.

## Discussion

For ET0 modeling, the simple and hybridized AIs have been examined in several studies (as mentioned in the introduction section). Mohammadi and Mehdizadeh^2^, Roy et al.^27^, Tao et al.^28^, Eslamian et al.^29^, Aghajanloo et al.^30^, Yin et al.^31^, and Gocić et al.^32^ are such these studies that have shown the combination of AIs with bio-inspired algorithms, can significantly improve the accuracy of simple AIs in ET0 modeling; which is similar to the current study’s results. However, in these mentioned studies the modeling was only applicable in the “estimation” of ET0 and is not examined for future “prediction” of ET0 rates; which distinguishes the mentioned studies from the current study. The desirability of the prediction accuracy of time series models in the current study is similar to the research of Gautam and Sinha^54^, Landeras et al.^15^, Psilovikos and Elhag^63^, Mossad and Alazba^64^, and Bouznad et al.^65^, that have been conducted in different climatic regions. The superiority of time series models over AIs in ET0 forecasting in Iran has also been reported in Ashrafzadeh et al.^18^ and Aghelpour and Norooz-Valashedi^19^. However, their studies only addressed the humid northern climate of Iran. Additionally, Ashrafzadeh et al.^18^ and Aghelpour and Norooz-Valashedi^19^ used non-hybridized artificial intelligence models, while the current research showed that the novel hybrid ANFIS-DE model can significantly increase the accuracy of the simple ANFIS model. In Brazil, however, AIs provided a relatively more accurate prediction of ET0 than time series models did (Lucas et al.^16^), which contradicts the results of the current study. This contradiction could be due to the differences between the climatic conditions of the studies’ regions.

Comparing the climates of the present study showed that the geographical location and the physical systems involved can be factors influencing the accuracy of ET0 prediction. For example, the humid regions of northern Iran are affected by Caspian atmospheric systems and various western systems, such as the Black Sea and the Mediterranean Sea, whereas the western and southwestern regions of Iran (like Shiraz and Ahwaz) are only weakly affected by the Saudi Arabia’s high-pressure and Sudan’s low-pressure systems. Susceptibility to a large number of systems can disrupt the order of time series, reduce autocorrelation, and consequently lead to poor prediction. This difference in the order of the ET0 series in different climates is depicted in the diagrams of Fig. 9. On the other hand, these three stations of Shiraz, Ahwaz, and Yazd, are located near the subtropical high-pressure belt (SHPB) (latitude 30 degrees), which can stabilize the weather regime in these areas, and thus make the ET0 series more regular. By moving away from the SHPB and approaching the latitudes of the humid northern regions, the effects of the irregularity of the annual regime become more obvious. This irregularity can decrease the autocorrelation of ET0 series (it is almost distinguishable in ACF plots of Fig. 5), and since the predictions are directly affected by ET0 time lags and autocorrelation within them, it can eventually cause a relative increase in the prediction errors in these humid areas.

## Conclusion

Studies have shown that the water requirement of plants can be predicted with outstanding accuracy by using the time lags of the evapotranspiration variable. The currently used data-driven approaches could provide acceptable predictions of ET0, regardless of the various atmospheric and physical factors that affect it. This result is similar in all currently studied climates. Despite the significant improvement of the ANFIS model combined with the differential evolution optimization algorithm (about 16%), it still fails to compete with the SARIMA linear model. According to Ashrafzadeh et al.^18,66^, the reason is that the linear autocorrelation is stronger than nonlinear autocorrelation in the ET0 time series. Finally, the present study proposes time series models for a better prediction of ET0 for two reasons: (1) higher accuracy and (2) the simplicity of use. Another important conclusion of this paper is that the climate type of a region significantly affects the accuracy of the models predicting ET0. ET0 was predicted more accurately in the arid and semi-arid climates of southern Iran than the humid and sub-humid regions of its north. Due to the high accuracy and promising results of the present study, using these data-driven models to predict plants’ water needs in other geographical areas is recommended. As a practical aspect of the current results, to predict the actual water requirement of a specific crop, the predicted ET0 rate can be obtained by multiplying the crop’s coefficient (FAO coefficients or the local reported coefficient). Moreover, utilizing the current models, especially SARIMA and the hybrid ANFIS-DE, has research value for long-term and multi-ahead years prediction of monthly ET0. The use and comparison of stochastic, artificial intelligence, and metaheuristic models in predicting ET0 on a daily scale can be an interesting topic of study, which we suggest to future researchers in this field. It’s worth mentioning that due to the limitations of the SARIMA model (which cannot consider other options as input to the model except the time lags of the evaporation variable itself), the machine learning models were applied by the inputs of ET0 time lags, to make a logicalcomparison. Therefore, it is suggested that future studies investigate the impacts of other hydro-meteorological factors’ time lags, such as droughts, heat waves, solar radiation, temperaturte, humidity, wind speed, etc. for ET0 prediction, which can only be applied by machine learning algorithms.

## Data Availability

The datasets used and/or analyzed during the current study are available from the corresponding author on reasonable request.
